# Similar Mechanisms of Movement Control in Target- and Effect-Directed Actions toward Spatial Goals?

**DOI:** 10.3389/fpsyg.2012.00539

**Published:** 2012-12-06

**Authors:** Andrea M. Walter, Martina Rieger

**Affiliations:** ^1^Max Planck Institute for Human Cognitive and Brain SciencesLeipzig, Germany; ^2^Institute for Psychology, University for Health Sciences, Medical Informatics and TechnologyHall in Tirol, Austria

**Keywords:** action targets, action effects, motor control, visual-spatial action goals, movement kinematics, ideomotor theory

## Abstract

Previous research has shown that actions conducted toward temporal targets and temporal effects are controlled in a similar way. To investigate whether these findings also apply to spatially restricted movements we analyzed movement kinematics of continuous reversal movements toward given spatial targets and toward self-produced spatial effects in two experiments. In Experiment 1 target- and effect-directed movements were investigated in three different goal constellations. A spatial target/effect was always presented/produced on one movement side, on the other side either (a) no target/effect, (b) the same target/effect, or (c) a more difficult target/effect was presented/produced. Results showed that both target-directed and effect-directed movements have a typical spatial kinematic pattern and that both can be equally well described by linear functions as suggested by Fitts’ Law. However, effect-directed movements have longer movement times. In Experiment 2 participants performed target-directed movements to the one side and effect-directed movements to the other side of a reversal movement. More pronounced spatial kinematics were observed in effect-directed than in target-directed movements. Together, the results suggest that actions conducted toward spatial targets and spatial effects are controlled in a similar manner. Gradual differences in the kinematic patterns may arise because effects are cognitively more demanding. They may therefore be represented less accurately than targets. However, there was no indication of qualitative differences in the cognitive representations of effects and targets. This strengthens our assumption that both targets and effects play a comparable role in action control: they can both be viewed as goals of an action. Thus, ideomotor theories of action control should incorporate action targets as goals similar to action effects.

## Introduction

Every day we perform intentional, goal-directed actions. Action goals differentiate an action from pure movement and fall into two broad categories. The goal of an action can either consist of generating a change in the environment (i.e., to produce an effect, for example turning on a switch in order to illuminate a dark room) or of changing one’s own situation in the environment (i.e., to move to a physical target, for example reaching out in order to grasp a cup). In the following we refer to these different types of goal-directed actions as effect-directed and target-directed actions, respectively.

Action goals have been known to play an important role in movement organization for a long time. In the present paper action goals are viewed in the light of the ideomotor theory of action control (James, 1890/1981; Prinz, 1997). The ideomotor theory has found broad empirical evidence (Elsner and Hommel, 2001, 2004; Hommel et al., 2003; for a historical overview see Stock and Stock, 2004) and states that an action is selected, initiated, and executed by anticipating the perceptual consequences of the action in question. Here we assume that both targets and effects are represented as action goals in motor control in the sense of the ideomotor theory. The representation of the intended perceptual consequences, in both target- and effect-directed movements, is responsible for the initiation, selection, and execution of a movement. In effect-directed actions the goal is the production of the effect and the manipulation of the environment itself. Target-directed actions also entail the representation of action goals such as “to be at a certain place at a given time.”

However, so far studies investigating predictions derived from ideomotor theory have mainly been concerned with the role of action effects. If action targets are considered at all, they are usually not treated as major goals of an action but as subgoals. For example, action targets are sometimes defined as the location at which an event has to occur (e.g., participants perform a key press in a certain location) before an effect occurs (e.g., an effect tone; Hoffmann et al., 2009). In this kind of situation targets and effects are related, and effects are higher in the goal hierarchy. In other terms, according to ideomotor theories, which distinguish between proximal (related more closely to the body) and distal (related to the environment) action effects (Prinz, 1987; Hoffmann et al., 2007), effects are more distal than targets in such experiments. Such a scenario applies of course to many everyday situations but not to all. As outlined above, it is not always the goal of an action to produce a change in the environment (to produce an effect), but it is also sometimes the goal to change one’s own situation in the environment (e.g., to move to a target). In the present study, we treated targets and effects as two different types of goals, which may be hierarchically equal and independent from each other. Thus, we designed the experiments in a way that the cognitive representations of targets and effects reside on the same level of “distality.” Participants moved to visuo-spatial targets and moved to produce visuo-spatial effects. In both instances, participants received the same proximal effects (i.e., proprioception, kinesthesis), but the distal goal representations differed. With effects, the distal goal representation consisted of the occurrence of the effect, whereas with targets the distal goal representation consisted of being in a certain position. Still, as both goal representations are major action goals, they should have a similar influence on movement execution.

Thus, the major goal of the present study was to investigate the commonalities and differences between target-directed and effect-directed actions and their underlying mechanisms of action control. Recently, we have shown that the same mechanisms of action control underlie movements directed toward auditory-temporal targets and auditors-temporal effects (Walter and Rieger, 2012). Walter and Rieger (2012) showed that typical temporal movement kinematics emerged when participants synchronized movements with regularly presented tones (target-directed movements) or produced tones themselves (effect-directed movements). We concluded that both targets and effects can be seen as goals of an action influencing movement execution by the anticipation of upcoming events. This study however only investigated auditory-temporal stimuli as action goals. In the present study, we wanted to investigate whether our previous conclusions extent to visual-spatial action goals. This is not self-evident, because differences in the way spatially and temporally restricted movements are controlled are observed in some studies (e.g., Heuer, 1993; Franz et al., 1996; Maslovat et al., 2011).

The role of visual-spatial targets for movement planning and initiation has been demonstrated. For example, people bring their hand in a position that may be uncomfortable at the beginning of a grasping movement but that will allow them to be in a comfortable posture that facilitates optimal control at the end of the movement (known as the end-state comfort effect, for a review see Rosenbaum et al., 2012). Further, if participants have initial information about a second target in a two-step movement sequence, but no information about the first target before the beginning of the sequence, movements are initialized faster than when they have no information about both targets in the sequence (Herbort and Butz, 2009). This finding is consistent with models of anticipatory movement planning that claim that in a movement sequence each step is planned in reverse order (Fischer et al., 1997) and confirms the assumption that upcoming targets are processed and movement execution toward them can be partially planned, resulting in faster movement initiation.

A wide variety of studies investigated the role of visual-spatial targets for movement execution. Over a century ago Woodworth described that it is impossible to be fast and accurate at the same time when moving toward a visual target (Woodworth, 1899). This limitation of the motor system known as speed-accuracy tradeoff has been mathematically described by Fitts (Fitts, 1954; Fitts and Peterson, 1964) showing that movement time (MT) increases linearly with task difficulty. Fitts specified task difficulty (index of difficulty: ID) as a function of target width and target distance (for a review and different ways to calculate ID see Plamondon and Alimi, 1997). This relation is widely known as Fitts’ Law and has inspired scientific research until today, especially in the field of human computer interface studies. Fitts’ Law holds for bimanual tasks as well as tasks performed by dyads (Mottet et al., 2001). Further, Fitts’ Law can be applied for translational as well as rotational movements (Stoelen and Akin, 2010) and has been studied intensively for distant aiming tasks with computer devices (Kopper et al., 2010). Whereas most studies investigated pointing and aiming with discrete tasks (for a review see Elliott et al., 1991), in some studies continuous tasks were used (e.g., Mottet et al., 2001). The kinematics of movements aimed at spatial targets frequently show asymmetric velocity profiles (Elliott et al., 2001). Specifically, movements toward spatial targets show a kinematic pattern that differs substantially from the kinematics of movements toward non-targets. Movements toward spatial targets reach peak velocity earlier and have relatively long MTs (Rieger, 2007). We will refer to this pattern as spatial movement kinematics in the following. Such spatial movement kinematics lead to prolonged time in the target area at the end of the movement. This additional time can be used to increase spatial accuracy (Novak et al., 2000; Elliott et al., 2001; Rieger, 2007).

Studies investigating the role of visual-spatial effects have mainly been conducted in the context of the ideomotor theory of action control (e.g., Hommel, 1993; Hommel et al., 2001; Kunde et al., 2007). It has been shown that participants respond faster if an action produces an effect that is spatially compatible with their response (action-effect-compatibility, e.g., Kunde, 2001). Kunde (2001) showed that in compatible conditions (e.g., a left hand key press produces a light flash on the left side of the monitor) responses are initiated faster than in incompatible conditions (e.g., the left hand key press produces a light flash on the right side of the monitor). The role of action effects has also been investigated when participants use tools for generating visual-spatial action effects. For example, when participants produce a rightward or leftward movement of a cursor on a display (that is a visual-spatial effect) by moving a steering wheel clockwise or counter-clockwise, movements are initiated faster when stimulus location (left-right tones) correspond to the direction of the produced effect (stimulus-effect-compatibility, Proctor et al., 2004). Similarly, mental rotations facilitate manual rotations when the direction of the visual effect is compatible with the mental rotation (Janczyk et al., 2012). Whereas many studies investigated the role of visual-spatial effects for movement selection and initiation the question of their role for movement execution is rarely addressed. In other domains, it has however been shown that effect anticipation also affects action execution (Kunde, 2003; Kunde et al., 2004).

To sum up, the existing literature on the role of visual-spatial targets and the role of visual-spatial action effects for movement control suggests that visual-spatial targets as well as visual-spatial effects may both serve as action goals in the sense of the ideomotor theory. To the best of our knowledge the role of visual-spatial targets and effects for action control has however not been systematically investigated in one study under comparable conditions when they reside on the same level of “distality.” This is what we did in the present study.

Even though targets and effects may both serve as action goals, physical targets and effects also have some features that make them clearly distinguishable from each other. Targets are externally generated and usually present in the environment before, during, and after the movement. Thus they can provide precise information for movement aiming and movement correction. In contrast, effects are only present in the environment after the movement has been executed (and often only for a limited amount of time) and their anticipatory representation relies solely on internal generation. As a consequence, memory and learning processes play a more prominent role in effect-directed than target-directed movements. Attention demands may also be higher in effect-directed movements than in target-directed movements, because in addition to other types of feedback the visual action effect has to be monitored in effect-directed actions. As a consequence, performing effect-directed in comparison to target-directed actions should be cognitively more demanding.

Thus, evidence suggests that movements toward spatial targets could be controlled in a similar way as movements toward spatial effects, as they are both goals of an action. Their different features could however also lead to differences in movement control. In the present study we wanted to investigate whether movements toward spatial targets and spatial effects are controlled in a similar way by comparing movements toward visual-spatial targets and movements toward self-produced visual-spatial effects. To this aim, we compared the kinematics of movements generating visual-spatial effects and the kinematics of movements toward visual-spatial targets. Participants performed continuous reversal movements on the medial-lateral axis. In target-directed movements they reversed their movement on constantly presented spatial targets, whereas in effect-directed movements they produced spatial stimuli themselves. We analyzed *how* target-directed and effect-directed movements are executed.

## Experiment 1

Participants performed continuous reversal movements on the medial-lateral axis. They were asked to move continuously back and forth and reverse their movements within black boxes that were constantly present during an experimental trial (target conditions) or were asked to move constantly back and forth and to produce black boxes in the same position as in target conditions when they reverse their movements. We analyzed *how* target-directed and effect-directed movements are executed.

**Figure 1:**
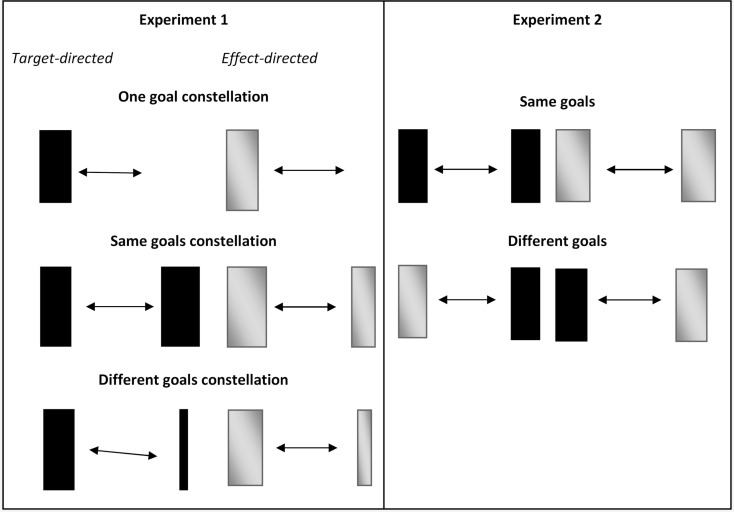
**Graphical overview of the goal constellations in Experiment 1 and Experiment 2**. Black boxes represent targets, gray boxes represent effects. Note that the color of targets as well as effects was black in the experiment. Wide boxes represent standard boxes (width: 2 cm, ID: 2.7), narrow boxes represent the more difficult boxes (width: 0.56 cm, ID: 4.3). In target-directed movements participants were asked to reverse their movements within constantly presented black boxes, while in effect-directed movements such boxes were self-produced as they only appeared whenever participants reached the *x*-position of the inner edge of the to-be-produced boxes.

Targets and effects were presented in three different goal constellations (see Figure 1, left panel). On one side of the movement always the same standard box was presented/to-be-produced. On the other side either (a) no box (one goal constellation), (b) the same standard box (same goals constellation), or (c) a different box with a higher Index of difficulty (different goals constellation) was presented/to-be-produced.

We expected that in the one goal constellation both target- and effect-directed movements toward the standard box show spatial kinematic patterns (early peak velocity, relatively long movement times) compared to movements toward the no box side. No such differences should be observable in the same goals constellations. In different goals constellation target-directed movements toward the more difficult box (Fitts, 1954) should show more pronounced spatial movement kinematics compared to movements toward the standard box. As we assume that both targets and effects can be viewed as goals of an action we expected to observe similar movement kinematics in target and effect conditions. We expected that effect-directed movements have higher spatial variability since the exact position of the effect is only seen at the endpoint of the movement and thus has to be remembered, which is cognitively more demanding. Nevertheless, we expected that Fitts’ Law (Fitts, 1954) can equally well describe target and effect conditions. The comparison of target- and effect-directed movements across goal constellations is of particular interest in order to investigate how the goal representations in target- and effect-directed movements are formed. Not only the presence/absence of a visual target is important for movement execution, but also its characteristics (i.e., target width). It is not clear, whether this will also be observed for self-produced visual effects. If only the presence/absence of a visual effect is represented but not its characteristics (width), movement kinematics in the same and different goals constellation should not differ in the effect condition (but they should differ from the kinematics in the one goal constellation). However, if the characteristics of the visual effect (width) are represented in effect conditions, movement kinematics in the same goals and different goals constellation should differ from each other, similar to what we expect in target conditions.

### Method

#### Participants

Twenty healthy participants (10 female) took part in this experiment. All of them were right-handed according to Edinburgh Inventory (Oldfield, 1971) with a mean laterality quotient of 91 (SD = 15). Their mean age was 25.6 years (SD = 2.4 years). All of them reported normal or corrected-to-normal vision. They gave informed consent prior to the experiment and received 7 Euro for participation.

#### Materials and apparatus

Movements were recorded with a 30.5 cm × 45.5 cm Wacom Ultrapad A3 writing pad at a resolution of 500 pixels per cm and at a rate of 172 Hz that was placed on a desk. Participants performed movements with their right (dominant) hand, which was shielded from view by a cover. Participants were able to see their movement trace consisting of a blue circle (4 mm in diameter) on a screen (17″, resolution: 1024 × 768 pixels, vertical refresh rate: 100 Hz). Movement distance on the writing pad equaled movement distance on screen. The screen was placed behind the pad at a distance of 60 cm from the participants and 9 cm higher than the pad. Spatial stimuli consisted of black boxes (distance between the centers 10.6 cm, standard width: 2 cm, ID = 2.7, more difficult width: 0.56, ID = 4.3) presented 5.3 cm to left and/or the right of the middle of the screen. If only one box was present a black line of 10.6 cm length aligned horizontally in the middle of the screen indicated the approximate length of a movement in a demonstration phase. A red box (0.5 cm × 0.5 cm) presented in the middle of the screen served as a starting box. The software Presentation 14.1 was used for stimulus presentation and data recording.

#### Procedure

The experiment took place in a dimly lit room. Participants were asked to perform continuous reversal movements on the medial-lateral axis without pausing at the reversal points. Movements were performed in two different goal conditions: target condition and effect condition. When performing target-directed movements, participants were asked to reverse their movements within constantly presented black boxes. When performing effect-directed movements, participants were asked to produce such boxes themselves. Before trials in the effect conditions started these black boxes were presented in an 8 s demonstration phase and participants were instructed to vividly keep the position and the width of the boxes in mind without moving. During experimental trials the box/boxes only appeared when participants reached the x-position of the inner edges of the (at this point in time not visible) boxes. In the instructions for the effect condition, participants were asked to produce such boxes of the same width and at the same position at their movement reversals. In both goal conditions, participants were asked to perform the task as fast and as accurately as possible.

At the beginning of the experiment participants received general instructions explaining all goal constellations and types of movements. Detailed instructions and visual stimuli were also presented on the screen before each trial. Participants started a trial themselves by entering the starting box, which appeared together with the instructions, with their pen whenever they were ready to begin. Trial duration was always 40 s.

Participants performed four training trials: two target condition trials and two effect conditions trials, each in the one goal constellation and the same goals constellation. The combination of three different goal constellations with two goal conditions, together with the balancing of the locations (left, right) of the standard box resulted in 12 experimental trials (in the same goals constellation the same number of trials as in the other constellations was conducted). Trials were presented in random order (restriction: not more than three trials of the same goal condition in a row). Participants completed three series of these 12 trials, after each of those series they had the opportunity to take a short break. The whole experiment took approximately 45 min.

#### Data analysis

Raw data were smoothed with a non-linear smoothing algorithm (Mottet et al., 1994) by using weighted and moving medians in a seven data point window. After that, pen velocity was determined at each measured point in time (i.e., every 5.8 ms) and then also smoothed with the same algorithm. The first 10 s of each trial were excluded from further analyses. For every goal condition in every goal constellation six trials were available for analysis. Since displacements on the *y*-axis were small (*M* = 0.29 cm, SD = 0.28 cm), only the maximum displacements on the *x*-axis were analyzed.

The reversal points (onsets and endpoints of a movement in one direction) were defined as the most leftward or rightward points of a movement followed by two data points indicating that the movement direction had changed. Movements were excluded from analysis if (a) participants did not move continuously (not more than 1 mm within the first 50 ms of a movement), (b) movement length was smaller than 5.3 cm (i.e., half of the instructed length of a movement), and (c) participants did not cross the middle line of the screen. Using these criteria less than 1% of movements were excluded from analyses in both target and effect conditions. A preliminary data analysis indicated that there were no differences in the data patterns between movements to the left and the right side. Therefore data were collapsed over this factor. The following statistical procedures were applied to both experiments: (a) if appropriate we report Greenhouse–Geisser corrected *F* values, (b) only higher order effects are reported if the lower order effects cannot be interpreted on their own, (c) significant effects were further analyzed using paired-sample *t*-tests, and (d) if appropriate Bonferroni corrected *p* values are reported.

The following set of dependent variables was analyzed in both experiments. To characterize the shape of trajectory, the time to reach peak velocity relative to the complete duration of the movement (proportional time to peak velocity in %, PTPV), and the time spent on one movement relative to the time spent on the complete reversal movement (proportional movement time in %, PMT) were analyzed. To characterize temporal performance the duration of a whole reversal movement (in ms, RMT) was analyzed. To characterize spatial performance the variability around the average endpoint of a movement (in cm, EP_V) and movement distance on the *x*-axis (in cm, Dist_X) were calculated. PTPV, PMT, and EP_V were analyzed using 3 × 2 × 2 repeated measurements analyses of variances (ANOVAs) with the factors GoalConstellation (one goal, same goals, different goals), GoalCondition (targets, effects), and BoxType (standard, manipulated). Note that “manipulated” in the factor Box Type can stand for no box (one goal constellation), the same standard box (same goals constellation), or the more difficult box (different goals constellation). RMT and Dist_X were subjected to 3 × 2 factors ANOVAs with the factors GoalConstellation (one goal, same goals, different goals) and GoalCondition (targets, effects), because those variables cannot be calculated separately for both sides of the reversal movement.

Furthermore, we calculated effective Index of Difficulty (eID) using effective target width (Welford, 1968; Zhai et al., 2004). In order to analyze whether the same amount of variance is explained by Fitts’ Law in target and effect conditions, we used eID and MT of every condition and computed correlations between eID and MT for every participant. The individual correlations were *z*-transformed (Fisher’s *z*-transformation). *t*-Tests were run on those transformed values. The average correlations reported here in the text are reconverted from the average Fisher’s *z*-values. We also calculated individual linear regression functions for each participant and each goal condition (target, effect) and used the estimated β values and intercepts for *post hoc* *t*-test analyses.

As our hypotheses partly consist of null-hypotheses (i.e., we expect no significant differences between target- and effect-directed movements) we calculated confidence intervals in order to assess whether differences between the two conditions are likely to be meaningful (Loftus, 1996). Confidence intervals for within-participant designs were calculated from normalized data according to Cousineau (2005), with the correction procedure suggested by Morey (2008). To gain further evidence for a functional similarity of target- and effect-directed movements we also calculated Pearson correlations between target and effect conditions for PTPV and PMT for each participant. Individual correlations were Fisher *z*-transformed and the average correlation coefficients reported here are reconverted from the average Fisher’s *z*-values.

### Results

#### Shape of trajectory

##### Proportional time to peak velocity

**Figure 2:**
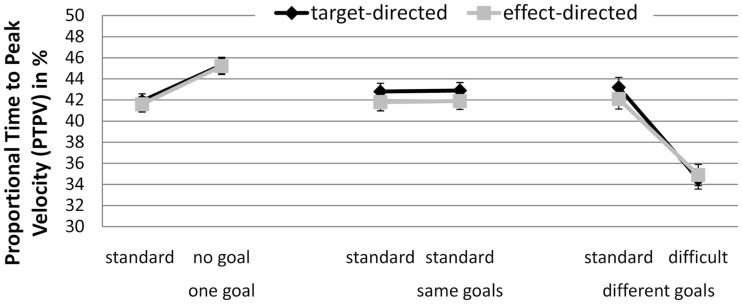
**Experiment 1: means and confidence intervals of Proportional Time to Peak Velocity in % (PTPV)**.

There was a significant interaction between GoalConstellation and BoxType, *F*(2, 38) = 17.16, *p* < 0.001, $\eta_{p}^{2}$ = 0.48 (see Figure 2). In the one goal constellation PTPV was lower when moving toward the standard box (*M* = 41.7%) than when moving away from it to the no box side (*M* = 45.3%). In the different goals constellation the opposite pattern was observed: when moving toward the more difficult box, PTPV was lower (*M* = 35%) than when moving toward the standard box (*M* = 42.7%). No such difference between the sides was observed in the same goals constellation. There were no significant main effect of and no significant interactions with the factor GoalCondition, indicating that effect- and target-directed movements were performed in a similar way. The average correlation between target conditions and effect conditions was high (*r* = 0.78) also pointing to a functional similarity between them.

##### Proportional movement time

**Figure 3:**
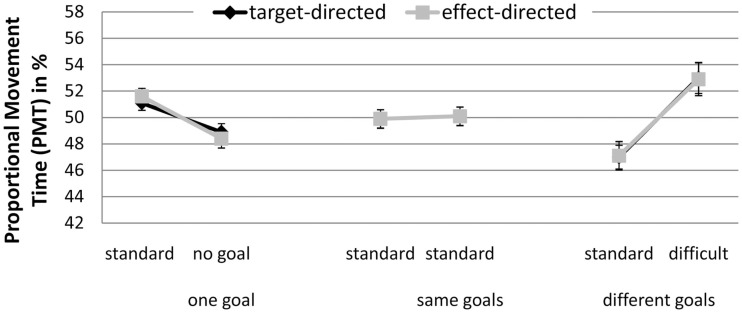
**Experiment 1: means and confidence intervals of Proportional Movement Time in % (PMT)**.

A significant interaction between GoalConstellation and BoxType, *F*(2, 38) = 10.94, *p* < 0.001, $\eta_{p}^{2}$ = 0.37 was observed (see Figure 3). In the one goal constellation PMT was higher for movements toward the standard box (*M* = 51.4) in comparison to movements to the no box side (*M* = 48.6%). The reverse pattern was observed in the different goals constellation. Here PMT toward the more difficult box was higher (*M* = 52.9%) than toward the standard box (*M* = 47.1%). No such difference between the sides was present in the same goals constellation. Again, there were no significant main effect of and no significant interactions with the factor GoalCondition. Further, again the average correlation between target and effect conditions was high (*r* = 0.89).

#### Temporal performance

##### Reversal movement time

There was a significant main effect of GoalConstellation, *F*(2, 38) = 13.84, *p* < 0.001, $\eta_{p}^{2}$ = 0.42. RMT in the one goal constellation (*M* = 1071 ms) did not differ significantly from RMT in the same goals constellation (*M* = 1137 ms), but RMT in the different goals constellation (*M* = 1415 ms) was significantly higher than in both other constellations (*p* < 0.05). This finding can be attributed to the presence of a more difficult spatial goal in this constellation than in the other constellations. A significant main effect of GoalCondition, *F*(1, 18) = 9.54, *p* < 0.006, $\eta_{p}^{2}$ = 0.33, indicated that RMT was higher in effect-directed movements (*M* = 1245 ms) than in target-directed movements (*M* = 1171 ms; see Table 1).

**Table 1:** **Experiment 1: variables describing temporal and spatial performance**.

	One goal		Same goals		Different goals	
	*M* (CI)		*M* (CI)		*M* (CI)	
**Reversal movement time in ms (RMT)**
Target-directed	1045 (391)		1103 (588)		1365 (892)	
Effect-directed	1097 (495)		1172 (601)		1465 (472)	

	**Standard**	**Manipulated**	**Standard**	**Manipulated**	**Standard**	**Manipulated**

**Endpoint variability in cm (EP_V)**
Target-directed	0.53 (0.1)	0.85 (0.1)	0.6 (0.1)	0.6 (0.1)	0.54 (0.1)	0.43 (0.1)
Effect-directed	0.56 (0.1)	0.82 (0.1)	0.57 (0.1)	0.59 (0.1)	0.56 (0.1)	0.51 (0.1)
**Movement distance on the *x*-axis in cm (Dist_X)**
Target-directed	10.8 (0.15)		10.9 (0.12)		10.9 (0.11)	
Effect-directed	10.6 (0.15)		10.8 (0.12)		10.9 (0.13)	

*Means and confidence intervals (in parenthesis) of Reversal Movement Time in ms (RMT), Endpoint Variability in cm (EP_V), and Movement Distance on the *x*-axis in cm (Dist_X)*.

#### Spatial performance

##### Endpoint variability

There was a significant GoalConstellation × BoxType interaction, *F*(2, 38) = 14.84, *p* < 0.001, $\eta_{p}^{2}$ = 0.44, that indicates that in the one goal constellation movements toward the side with the standard box (*M* = 0.54 cm) had a lower EP_V than movements to the no box side (*M* = 0.84 cm; see Table 1). In contrast, in the different goals constellation lower EP_V was observed in movements toward the more difficult box (*M* = 0.47 cm) in comparison to movements toward the standard box (*M* = 0.55 cm; all *p* < 0.05).

##### Movement amplitude on the *x*-axis

There was a main effect of GoalCondition, *F*(1, 19) = 5.9, *p* < 0.025, $\eta_{p}^{2}$ = 0.24. Target-directed movements (*M* = 10.9 cm) had higher MA than effect-directed movements (*M* = 10.7 cm; see Table 1).

##### Functions according to Fitts’ Law

**Figure 4:**
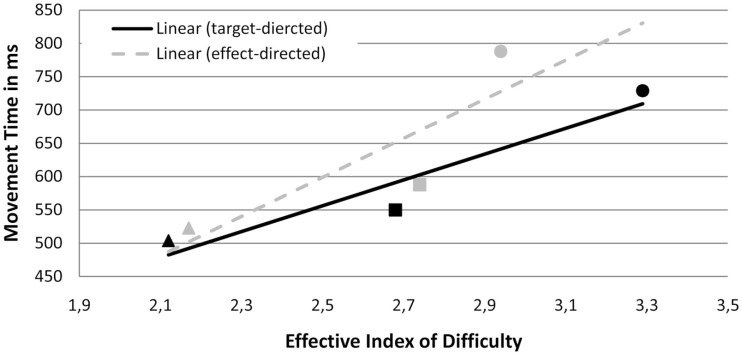
**Experiment 1: means and linear functions of the relation between effective Index of difficulty (eID) and movement time (MT in ms) for target-directed and effect-directed movements toward the manipulated goal**. Triangles symbolize the one goal constellation, squares the same goals constellation, and circles the different goals constellation. Black markers indicate target conditions, gray markers indicate effect conditions.

The correlation eID and MT was *r* = 0.30 in the effect conditions and *r* = 0.38 in the target condition (see Figure 4). These correlations did not significantly differ from each other, *t*(19) = 1.12, *p* > 0.05, indicating that the amount of variance explained by a linear relationship between eID and MT did not significantly differ between both types of movement. Fitting functions were also similar: β values, *t*(19) = −0.74, *p* > 0.05, and intercepts, *t*(19) = 0.82, *p* > 0.05, did not significantly differ between the target condition [*R*^2^ = 0.46, *p* < 0.05; *M* (β) = 208, SD = 160; *M* (intercept) = 41, SD = 341] and the effect condition [*R*^2^ = 0.54, *p* < 0.05; *M* (β) = 302, SD = 514; *M* (intercept) = −319, SD = 1850].

#### Discussion

We conducted Experiment 1 in order to find out whether similar mechanisms of action control underlie movements toward presented visual-spatial targets and self-produced visual-spatial effects. Overall the data show that the movement kinematics are very similar in target- and effect-directed actions. We observed no main effect of GoalCondition and no interactions with the factor GoalCondition in PTPV and PMT. Both movement types can be equally well described by a linear Fitts’ function, and the functions were not significantly different from each other. Moreover, no differences in EP_V between both movement types were observed. A typical relative spatial kinematic pattern was obtained in the one goal constellation: when moving toward the standard box PTPV was lower and PMT was higher than when moving to the no box side. This pattern reverses in the different goals constellation: here PTPV was lower and PMT was higher when moving toward the manipulated (more difficult) box side than when moving toward the standard box side. Spatial variability as described by EP_V follows the same pattern: in the one goal constellation movements toward the manipulated box side (no box) have higher EP_V, in the different goals constellation movements toward the standard box side have higher EP_V. In the different goal constellation movements have also a longer RMT. Small differences between target-directed and effect-directed movements were also obtained. Effect-directed movements have higher RMT and smaller movement amplitudes on the *x*-axis than target-directed movements.

As expected, target-directed and effect-directed movements are performed in a similar way. When comparing movements toward a spatial goal with movements toward a side without a goal a typical spatial kinematic pattern (low PTPV, high PMT) emerges no matter if aiming toward a spatial target or producing a spatial effect. For both types of movement it can therefore be assumed that this kinematic pattern reflects the specific goal characteristics (here: spatial characteristics) and helps to achieve the goal of the movement (to perform movements spatially accurate). It has been speculated that the additional time in the target area at the end of the movement helps to improve spatial accuracy (Novak et al., 2000; Elliott et al., 2001; Rieger, 2007). Another hint for this assumption comes from studies showing that the skewness in velocity profiles increases as spatial accuracy demands increase and/or targets are small (Hogan and Flash, 1987; MacKenzie et al., 1987; Helsen et al., 1998; Elliott et al., 2001). Whereas this kinematic pattern has previously been observed in studies in which target-directed movements were investigated (Elliott et al., 2001; Rieger, 2007), we were able to demonstrate that it also occurs with effect-directed movements. The observation that both target-directed and effect-directed movements can be equally well described by a linear Fitts’ function, and that the functions do not significantly differ from each other, also points to a functional similarity of both as goals of an action. Surprisingly, no differences in EP_V between both movement types were found. Thus, even though participants have to remember location and width in effect conditions they seem to fulfill this task quite well. In the different goals condition they show lower EP_V toward the more difficult goal side in both conditions. This result, together with the data on the shape of the trajectories suggests, that participants do not only represent target location but also target width in effect conditions.

Differences between both types of movement were also found: effect-directed movements have higher RMT and slightly shorter amplitudes (0.2 cm) than target-directed movements. Thus, even though the general movement *pattern* is the same as in target-directed movements, the data also point to differences between targets and effects. Those differences probably arise from higher cognitive demands in effect conditions: the need to remember the location of the effects, which may result in less precise goal representations. Those less precise goal representations may be compensated by longer reversal movement times and slightly shorter amplitudes.

To sum up, target-directed and effect-directed movements seem to be controlled in a similar manner. Movement execution is thereby influenced by the upcoming goal *before* the effect appears or the target is reached, indicating that goal anticipations are important for the way how a movement is executed. Differences between target- and effect-directed actions can be attributed to higher cognitive demands in effect conditions.

## Experiment 2

Results of Experiment 1 indicated that spatial kinematics are comparable in target- and effect- directed movements to visual-spatial goals, pointing to similarities in their control mechanisms. However, data also indicated that effects are represented less precisely, probably due to higher cognitive demands. Whereas in Experiment 1 we compared movements toward targets and effects performed in different trials, in Experiment 2 we combined target-directed and effect-directed movements within trials (a target on one side of the reversal movement, an effect on the other side of the reversal movement). We expected that a direct comparison of target- and effect-directed movements within one trial may enhance differences between them. When participants are asked to move to targets and effects within one goal constellation, one of those goals may be dominant (i.e., result in a more pronounced representation) over the other goal. Further, this setup prevents that participants move at different overall speed levels and also prevents shorter MAs in effect-directed than in target-directed movements (as it was the case in Experiment 1).

Participants again performed continuous reversal movements on the medial-lateral axis to visual-spatial goals. There were four conditions: (a) target-directed movements on both reversal sides, (b) effect-direct movements on both reversal sides, (c) target-directed movements to the left side and effect-directed movements to the right side, and (d) target-directed movements to the right side and effect-directed movements to the left side.

Our hypotheses concerning the conditions with different goals on both sides of the reversal movement were undirected. On the one hand, the goal representation for the spatial target may be more pronounced than for the spatial effect, because the target is constantly visible. If this is the case, a more pronounced spatial kinematic pattern for the target side should be observed (higher PMT, lower PTPV in target-directed movements). On the other hand, as effect conditions seem more difficult, participants may devote more of their cognitive resources to the effect and thus, the effect representation may be more pronounced than the target representation. If this is the case, effect-directed movements should show a more pronounced spatial kinematic pattern (higher PMT, lower PTPV in effect-directed movements). We further expected, based on the results of the same goals constellation condition in Experiment 1, that no differences in movement kinematics between targets and effects occurs when the same type of movement is conducted toward both sides.

### Method

#### Participants

Twenty healthy participants (11 female; mean age = 23.7 years, SD = 3.0) took part. According to the Edinburgh Inventory (Oldfield, 1971) all of them were right-handed (mean laterality quotient = 94, SD = 10). All of them reported normal or corrected-to-normal vision. They gave informed consent and received 7 Euro for participation. None of them had participated in Experiment 1.

#### Materials and apparatus

The experimental setup was the same as in Experiment 1. Therefore only differences are reported here. Visual stimuli consisted the standard boxes of Experiment 1 (black boxes, width: 2 cm, height: 9 cm, ID = 2.7, presented 5.3 cm to left and to the right of the middle of the screen).

#### Procedure and design

Visual-spatial goals were presented in four different goal combinations: two with same goals which were (a) target-directed movements on both reversal sides (target condition), and (b) effect-direct movements on both reversal sides (effect condition), and two with different goals which were (c) target-directed movements to the left and effect-directed movements to the right side, and (d) target-directed movements to the right and effect-directed movements to the left side (see Figure 1, right panel).

As in Experiment 1 participants were instructed to perform target-directed and effect-directed movements. In conditions in which targets and effects were combined participants were asked to reverse the endpoints of their movements within the constantly presented black box on one side. When performing effect-directed movements, participants were asked to produce such boxes themselves as in Experiment 1. Each condition was preceded by instructions and an 8 s demonstration phase of the widths and positions of the boxes. Participants were instructed to keep those vividly in mind and to produce them in the effect conditions during the experimental trials. Trial duration was always 40 s.

Each of the four goal combinations was conducted five times resulting in 20 experimental trials. Before the experimental trials were conducted participants performed four training trials, one in each condition. Trials were presented in random order with the exception that not more than three trials of the same condition were performed consecutively.

#### Data analyses

Data preparation was conducted as in Experiment 1. The first 10 s of the each experimental trial were excluded from further analyses. As again displacements on the *y*-axis were small (*M* = 0.43 cm, SD = 0.41 cm) only displacements on the *x*-axis were analyzed. The same exclusion criteria as in Experiment 1 were applied, leading to exclusion rates of less than 1% in each condition. Because the data patterns for movements to the left and right side were similar, data were collapsed over this factor. PTPV and PMT were analyzed using 2 × 2 repeated measurement ANOVAs with the factors GoalConstellation (same goals, different goals) and GoalCondition (targets, effects). RMT and Dist_X were subjected to ANOVAs with the factor GoalConstellation (same targets, same effects, different goals).

### Results

#### Shape of trajectory

##### Proportional time to peak velocity

**Figure 5:**
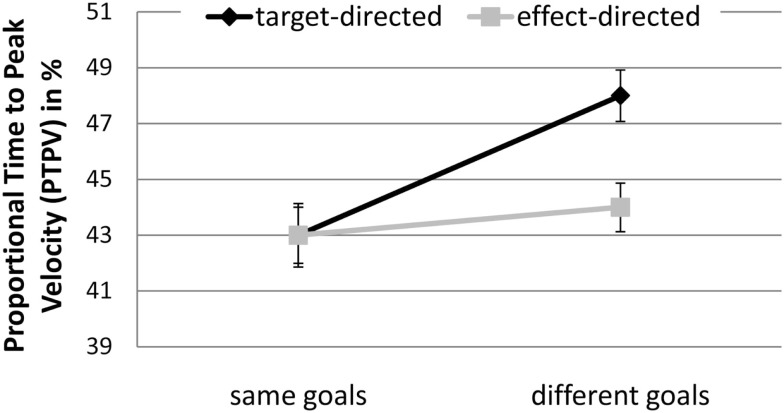
**Experiment 2: means and confidence intervals of Proportional Time to Peak Velocity in % (PTPV)**.

There was a significant interaction between GoalConstellation and GoalCondition, *F*(1, 19) = 12.1, *p* < 0.003, $\eta_{p}^{2}$ = 0.34. In the same goals constellation target- and effect-directed movements did not significantly differ in PTPV, whereas in the different goals constellation PTPV was significantly lower for effect-directed (*M* = 44.5%) than for target-directed (*M* = 48.1%; *p* < 0.05) movements (see Figure 5).

##### Proportional movement time

**Figure 6:**
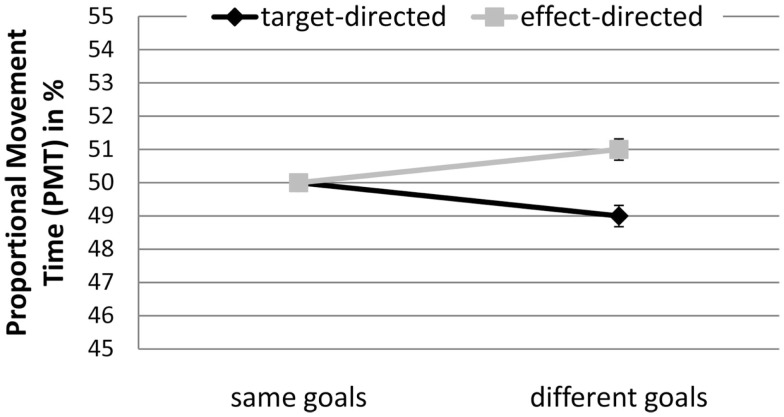
**Experiment 2: means and confidence intervals of Proportional Movement Time in % (PMT)**.

A significant interaction between GoalConstellation and GoalCondition, *F*(1, 19) = 8.0, *p* < 0.011, $\eta_{p}^{2}$ = 0.3, indicated that target-directed movements (*M* = 49%) had lower PMT than effect-directed movements (*M* = 51%) in different goals constellation, whereas no difference between the two types of movement was observed in same goals constellation (see Figure 6).

#### Temporal performance

##### Reversal movement time

The main effect of GoalConstellation was significant, *F*(2, 38) = 4.1, *p* < 0.024, $\eta_{p}^{2}$ = 0.18. Results were intransitive, only reversal movements in the same effects constellation took significantly longer (*M* = 947 ms) than movements in the different goals constellation (*M* = 809 ms, *p* < 0.05), whereas movements in the same targets constellation did not significantly differ from the other two conditions (see Table 2).

**Table 2:** **Experiment 2: variables describing temporal and spatial performance**.

Same targets *M* (CI)	Same effects *M* (CI)	Different goals: targets *M* (CI)	Different goals: effects *M* (CI)
**Reversal movement time (RMT)**			
895 (30)	947 (31)	809 (21)	
**Endpoint variability in cm (EP_V)**			
0.58 (0.006)	0.57 (0.006)	0.57 (0.005)	0.55 (0.006)
**Movement distance on the *x*-axis in cm (Dist_X)**			
10.8 (0.12)	10.7 (0.12)	10.8 (0.12)	

*Means and confidence intervals (in parenthesis) of Reversal Movement Time in ms (RMT), Endpoint Variability in cm (EP_V), and Movement Distance on the *x*-axis in cm (Dist_X)*.

#### Spatial performance

##### Endpoint variability

There were no significant main effects or interactions (see Table 2).

##### Movement distance on the *x*-axis

There were no significant main effects or interactions, showing that participants moved comparable distances in all conditions (see Table 2).

### Discussion

In order to enhance differences between effect-directed and target-directed movements, they were executed within the same reversal movement in one of the goal constellations of Experiment 2. Results of variables describing the shape of trajectory show that a more pronounced spatial kinematic pattern emerged in the different goals constellation toward effect-directed movements (lower PTPV, higher PMT). As expected, no significant differences were found in the same goals constellation. However, in the same effects constellation higher RMT were observed than in the different goals constellation. No significant effects were found in variables describing the spatial performance (EP_V and Dist_X).

As expected, based on the results of Experiment 1, no significant differences in shape of trajectory between target and effect conditions in the same goals constellation were observed. This provides further evidence for the functional equivalence of targets and effects as action goals. Interestingly, combining target- and effect-directed movements in one reversal movement enhanced differences between them: a more pronounced spatial kinematic pattern for effect-directed in comparison to target-directed movements was observed. Results of Experiment 1 suggested that effects have a less precise internal representation than targets. Thus, not the goal information provided by the experimental context (more precise in targets than in effects), but rather the cognitive resources devoted to the goal (more effortful for effects than targets) results in a more pronounced goal representation. This is in line with assumptions that movement kinematics are chosen in order to fulfill the task goals as well as possible (Rieger, 2007). In the same effects constellation significantly higher reversal movement time was observed, again underpinning the assumption that effects are represented less precise and are therefore more difficult to perform, which is then compensated with higher reversal movement time.

In summary, results of Experiment 2 again indicate that targets and effects are represented as action goals. However, less precise representation of effects is compensated by devoting more cognitive resources to effects, resulting in a more pronounced spatial kinematic pattern.

## General Discussion

We conducted the present study in order to investigate whether spatial targets and spatial effects play a comparable role in action control as action goals. This was done by analyzing *how* participants execute movements toward visual-spatial targets and visual-spatial effects. In two different experiments participants performed continuous reversal movements toward targets, effects or no goals. In Experiment 1 target-directed and effect-directed movements were compared across conditions in three constellations with varying goal features. In Experiment 2 both movement types were combined within one condition to enhance differences between them. Results indicated that the same mechanisms of action control underlie movements toward targets and effects, and that they are therefore equally represented as action goals. When compared across conditions no significant differences between targets and effects were observed in the shape of the trajectory (Experiment 1, and Experiment 2, same goals constellation) and in spatial variability (Experiment 1 and 2). Further, target- and effect-directed movements both show a more pronounced spatial kinematic pattern toward a goal than toward a no-goal (Experiment 1, one goal constellation). Similarly, both show a more pronounced spatial kinematic pattern toward a more difficult than toward an easier goal (Experiment 1, different goals constellation). In addition, both target-directed and effect-directed movements can be equally well described by Fitts’ Law (Experiment 1). Differences between target- and effect-directed movements were observed when compared within conditions. Here effect-directed movements showed a more pronounced spatial kinematic pattern (Experiment 2). Effect-directed movements require that participants remember the effect location and use the remembered information to plan, initiate, and execute their aiming movement. To compensate for this less precise representation participants devote more cognitive resources to the effects. The higher cognitive demands also result in longer reversal movement times toward effects (Experiment 1, and Experiment 2, same effects constellation).

One may argue that participants simply produced repetitive movements of similar amplitudes toward the same locations in both, target and effect conditions. We intentionally designed target and effect conditions as similar as possible, as we wanted to avoid that other differences in the characteristics of targets and effects (apart from being a target or a effect) can account for the results. Thus, targets and effects only differed in one decisive aspect: targets did not depend on the action of the participant (i.e., they were always visible), whereas effects dependent on the action of the participant (i.e., appeared when participants reached the target area). As the target stimulus and the effect stimulus were physically the same, and due to experiencing the stimulus as a target in 50% of trials, one may be concerned that participants’ experience of the effect as being self-produced may be reduced. This may have been the case if participants had repeatedly switched between target and effect conditions. However, in our experiments one trial always lasted for 40 s, which resulted in a stable current context (target or effect context) for the stimulus. Moreover, when combined within one trial (Experiment 2) differences between target-directed and effect-directed movements were enhanced. This indicates that participants indeed experienced target and effect conditions as different.

One may also be tempted to compare the visual effects in our study with what is termed visual feedback in other studies (e.g., Saunders and Knill, 2004; Roerdink et al., 2005; Thaler and Goodale, 2011). From a theoretical viewpoint, this is valid, because feedback certainly is an action effect. However, action effects in our study (appearance of a visual stimulus) were operationalized as the major goal of one reversal movement. In other studies investigating visual feedback the main purpose of a task is often not to “produce” the visual stimulus, but the visual feedback provides additional information about the current position. In addition to visual effects, participants also received visual feedback in our study: their current movement position was represented as a blue dot on the screen. Even though “effects” and “feedback” theoretically represent action effects, one may thus argue that the visual effects in our study (appearance of the boxes) reside on a higher level in the goal hierarchy of the task than visual feedback (cursor representing the current hand position), as it is the main purpose of the movement (or more specifically: the endpoint of the movement) to produce the effect which thus is the distal goal representation. It should be noted that in target conditions, participants also received visual feedback (cursor representing the current hand position). In target and effect conditions participants also received the same proximal effects/feedback (i.e., proprioceptive, kinesthetic). However, in target conditions participants received no visual effect. Rather, here the distal goal representation was to be at a certain position at a certain time.

Our results support the assumption that effect-directed movements are more difficult due to higher cognitive demands and that this is compensated by devoting more cognitive resources toward effects leading to a pronounced spatial kinematic pattern toward them. In line with this assumption are findings which indicate that (perceived) task difficulty influences movement kinematics. For example, Park and Kim (2008) manipulated target-size and movement amplitudes in a Fitts’ task separately such that both manipulations resulted in the same indices of difficulty. They investigated self-terminated horizontal elbow-extension movements. The authors found different mechanisms of movement control leading to an increase of MT in both conditions. In the target-size condition a decrease in triceps and biceps muscle activation, and a decrease in movement velocity with increasing index of difficulty was observed in both, the acceleration and the deceleration phase. In the movement amplitude condition triceps activation after movement onset and biceps activation during deceleration increased with increasing index of difficulty, resulting in a higher peak velocity, even though MT also increased with increasing index of difficulty. Thus, they conclude that perceived task difficulty influences movement control, but not *de facto* task difficulty (held constant across conditions). Further, in a spatial aiming task reaction time and MT to a first target increased as a function of the number of elements only when either the full response or the number of elements that have to be performed were specified in advance of the starting stimulus (Khan et al., 2007). Khan et al. conclude that when the number of to be performed elements is known in advance more complex movement integration strategies are preprogramed, which leads to increased executive control and in turn results in longer reaction times as well as longer MTs. Along these lines we assume that higher cognitive demands in effect-directed movements are compensated by devoting more cognitive resources toward effects. This results in a more careful strategy of movement execution and leads to a more pronounced spatial kinematic pattern in effect-directed movements when they are combined with target-directed movements.

Besides that effect-directed movements are more difficult to perform, the here presented experiments show that both target-directed and effect-directed movements show a typical spatial kinematic pattern toward visual-spatial goals. We take this as evidence that both targets and effects can be viewed as goals of an action. In the case of effects the goal of the action is the production of the effect itself and in the case of targets the goal is “to be at a certain place.” We assume that the representation of these goals shapes movement kinematics in the observed typical manner. As these goal representations are being formed *before* the movement is actually conducted and then influence its execution this is in accordance with ideomotor principles of action control, claiming that the anticipation of the intended consequences of an action influences movement selection (Knuf et al., 2001), initiation (Kunde, 2003), and also movement execution (Kunde et al., 2004). So far ideomotor theories mainly deal with action effects as action goals. Besides the possibility that proximal effects are produced at action targets (e.g., tactile sensations or sensations related to body postures) targets are neglected. In contrast, our study shows that both targets and effects may equally serve as action goals, evoking visual-spatial event anticipations. Ideomotor theories should thus be expanded to cover goal-based (including target- and effect-based), rather than only effect-based action control.

Both the here presented study and our study conducted with auditory-temporal goals (Walter and Rieger, 2012) show that the same mechanisms of action control underlie movements toward targets and effects as they can both be seen as goals of an action. This comparable result presented here is not obvious, as differences in the way spatially and temporally restricted movements are controlled are observed in some studies (e.g., Heuer, 1993; Franz et al., 1996; Maslovat et al., 2011). The findings of Walter and Rieger (2012) as well as the current study indicate that the equivalence of targets and effects as action goals holds for spatially as well as temporally restricted movements. This may also be the case in other modalities.

Note that the interpretation of our data relies partly on non-significant results. However, traditional null hypothesis testing does not tell us the probability that the null hypothesis is true (Cohen, 1994). Thus, drawing strong conclusions from non-significant results may be problematic. However, the very small confidence intervals, which indicate that the true deviation from H0 is unlikely to be large, an *a priori* hypothesized pattern in the data, and the high average correlations between target and effect conditions in the variables describing the shape of the trajectory in Experiment 1 render our explanation, that similar mechanisms of action control underlie target- and effect-directed actions, very likely.

Besides this general similarity in spatially and temporally restricted movements there is also a difference in the results from both studies: combining targets and effects within one reversal movement increased differences between effect- and target-directed movements toward spatial goals in the present study, whereas the same manipulation enhanced similarities between effect- and target-directed toward temporal goals in the previous study (Walter and Rieger, 2012). A reason for this can be that spatial targets and effects and temporal targets and effects may pose different demands on the cognitive-motor system. Spatial targets can be perceived all the time during a movement, whereas spatial effects cannot. In contrast, temporal targets and effects both only occur for a limited amount of time. Updating of timing in temporal targets can only occur at those points in time, whereas updating of the position of spatial targets can occur at any time. Thus, temporal targets and effects may be more alike in their degree of difficulty than spatial targets and effects. Consequently, when combined within one condition differences between temporal targets and effects are diminished as their similarity is then emphasized, whereas differences between spatial targets and effects are enhanced as they become more obvious, resulting in a more pronounced spatial kinematic pattern toward effects.

To conclude, movement kinematics toward spatial targets and spatial effects are shaped in a typical manner showing that both targets and effects can equally serve as action goals. Moreover, both target-directed and effect-directed movements can be described by Fitts’ Law in a similar manner. Only small differences are found between target-directed and effect-directed actions. When combined within one condition more cognitive resources are devoted to effect-directed than to target-directed movements leading to a more pronounced representation of effects. The influence of the anticipation of upcoming events on movement execution is in accordance with ideomotor theories of action control. Ideomotor theories should be expanded to include action targets as action goals similar to action effects and consequently cover goal-based, rather than effect-based action control.

## Conflict of Interest Statement

The authors declare that the research was conducted in the absence of any commercial or financial relationships that could be construed as a potential conflict of interest.
